# Cognitive or Cognitive-Motor Executive Function Tasks? Evaluating Verbal Fluency Measures in People with Parkinson's Disease

**DOI:** 10.1155/2017/7893975

**Published:** 2017-08-20

**Authors:** Alessandra Ferreira Barbosa, Mariana Callil Voos, Janini Chen, Debora Cristina Valente Francato, Carolina de Oliveira Souza, Egberto Reis Barbosa, Hsin Fen Chien, Letícia Lessa Mansur

**Affiliations:** ^1^Physical Therapy, Occupational Therapy and Speech Therapy Department, Faculty of Medicine, University of São Paulo, São Paulo, SP, Brazil; ^2^ReMove, Rehabilitation in Movement Disorders Research Group, University of São Paulo, São Paulo, SP, Brazil; ^3^Movement Disorders Clinic, Department of Neurology, Clinics Hospital, Faculty of Medicine, University of São Paulo, São Paulo, SP, Brazil

## Abstract

**Introduction:**

Executive function deficits are observed in people with Parkinson's disease (PD) from early stages and have great impact on daily living activities. Verbal fluency and oral diadochokinesia involve phonarticulatory coordination, response inhibition, and phonological processing and may also be affected in people with PD. This study aimed to describe the performance of PD patients and an age- and education-matched control group on executive function, verbal fluency, and oral diadochokinesia tests and to investigate possible relationships between them.

**Methods:**

Forty people with PD and forty controls were evaluated with Trail Making Test (TMT, executive function) and phonemic/semantic verbal fluency and oral diadochokinesia (/pataka/) tests. Groups were compared by ANOVA and relationships were investigated by Pearson tests.

**Results:**

People with PD showed longer times in parts A and B of TMT. They also said fewer words in phonemic/semantic verbal fluency tests and less syllables in the diadochokinesia test. Oral diadochokinesia strongly correlated to parts A and B of TMT and to phonemic verbal fluency.

**Conclusion:**

Oral diadochokinesia was correlated to executive function and verbal fluency. The cognitive-motor interaction in verbal fluency and oral diadochokinesia must be considered not to overestimate the cognitive or motor impairments in people with PD.

## 1. Introduction

People with Parkinson's disease (PD) experience nonmotor symptoms, such as attention/memory deficits and visuospatial disorganization [1]. Executive function plays an important role in these aspects and involves mental flexibility, decision making, problem solving, motor sequencing/inhibiting, and task switching [2]. The efficiency in daily living activities relies on the integrity of executive function and deficits can be found even in early stages of PD [3].

The incidence of mild cognitive impairment reaches 19–38% of people with PD [2] and may cause disability in self-caring, driving, and interacting [4] and increased falls risk [5, 6]. Executive function depends on frontal structures, which are impaired in people with PD, due to dopamine depletion in nigrostriatal projections [1, 7]. Deficits in executive function can be attributed to the reduced activity in the caudate nucleus, even in people without dementia [8].

Cortical cholinergic denervation is associated with cognitive decline in people with PD. Bohenen et al. (2015) investigated the relationship between cognitive function and imaging analysis [9]. They concluded that dopaminergic caudate nucleus denervation is frequent in people with mild cognitive impairment. Cognitive impairment progresses as cholinergic denervation increases. They also reported that the cholinergic system is probably overactivated in the initial phase of mild cognitive impairment, as a compensatory mechanism for dopaminergic denervation.

Several tests can be used to evaluate executive function, for example, Trail Making Test (TMT) and verbal fluency tests. TMT consists in drawing a trail to match numbers in sequence (part A) and number and letters alternately (part B). The time spent in part A reflects motor performance. Part B requires mental flexibility, task switching, response inhibition, and working memory and evaluates cognitive-motor dual-task performance [10]. TMT delta (time in part B − time in part A) is considered as a pure cognitive measure, because it isolates the cognitive impact added by alternating the letters sequence [10].

Verbal fluency tests are recommended for cognitive screening in people with PD. Although methods may vary [11, 12], participants are usually instructed to say as many words as possible in 60 seconds. In the phonemic test, words must begin with a determined letter. In the semantic test, words must belong to the same category. Most studies only interpret the score on fluency tests as a cognitive measure and do not consider the task motor demands. As people get older, speech production can be impaired. This loss can be attributed to a reduction in muscular strength, endurance, and coordination, which are intensified by PD [13].

Speech evaluation protocols include oral diadochokinesia tests that consist of rapid repetition of a syllable or syllable sequences as quickly as possible [14]. The decrease in the syllable production rate can be related to motor control, and speed reduction may be associated with maintaining intelligibility [15].

Verbal fluency tests can be considered cognitive-motor tasks. They involve phonarticulatory coordination, response inhibition, and phonological processing. Therefore, we hypothesized that the performance on verbal fluency would show higher correlation with part B of TMT (which is also a cognitive-motor measure) than with part A of TMT (motor measure) and delta TMT (cognitive measure). We also hypothesized that oral diadochokinesia would show higher correlations with part A of TMT (motor measure) than with part B of TMT (cognitive-motor measure) or delta of TMT (cognitive measure). This study aimed to describe the performance of people with PD, compared with an age- and education-matched control group, on executive function, phonemic/semantic verbal fluency, and oral diadochokinesia tests and to investigate possible relationships between these measures, due to cognitive-motor interactions.

## 2. Method

This study was approved by the Committee on Research Ethics at Clinics Hospital of University of São Paulo (process 1.631.497). All participants read and signed the written informed consent.

### 2.1. Participants

Seventy-eight outpatients with idiopathic PD, from the Movement Disorders Clinic of Clinics Hospital were invited to participate in the experimental group. Fifty-two volunteered and forty met inclusion criteria. Seven were excluded because they were in the early stage of the disease (Hoehn and Yahr score below 2). Five were excluded because they were adapting to recent changes on medications. Fifty-nine healthy older adults from a senior center of University of São Paulo were invited to participate in the control group. Forty-five volunteered and forty met inclusion criteria. Four controls were excluded for having less than four years of formal education. One control was excluded due to having a neurological condition.

People aged 50–79 years, with four or more years of formal education and Mini-Mental State Examination score above 23 [16], were included. Additional inclusion criteria for patients with PD were having received the diagnosis of PD according to the United Kingdom Parkinson's Disease Society Brain Bank criteria [17], Hoehn and Yahr [18] score of 2-3, and optimized daily dosage of antiparkinsonian drug treatment during the last four weeks prior to study entry. People with PD were on their best “on” state during assessment. Volunteers with acute/terminal illnesses, myocardial infarction in the last six months, moderate/severe chronic obstructive pulmonary disease, and neurological and/or muscular diseases (evaluated by self-report) were excluded.

Demographic data from both groups are described in Table 1.

### 2.2. Assessment

Participants were assessed individually in a fifty-minute session. The initial anamnesis consisted of collecting demographic/screening information (age, number of years of formal education, Mini-Mental State Examination score, and motor section of Unified Parkinson's Disease Rating Scale [17]). Then, participants were assessed with TMT, phonemic/semantic verbal fluency test, and oral diadochokinesia test. Tests were performed in random order to avoid learning effects. Participants were comfortably seated on a desk during evaluation.

In part A of TMT, participants connected circles with the numbers 1–25 in sequence. In part B, participants connected circles in a sequence with alternated numbers and letters (1-A-2-B-3-C-4-D-5-E-6-F-7-G-8-H-9-I-10-J-11-K-12-L-13). When errors occurred, the examiner said that there was an error and asked the participant to return to the last correct circle. The scores were the duration taken to complete each part. The test was interrupted if not completed within 300 seconds, and the highest possible score (300) was given [10].

In the phonemic verbal fluency test, participants were instructed to say words beginning with the letter F. In the semantic verbal fluency test, participants were instructed to say out loud as many animals as they could remember in 60 seconds. Scores were calculated by counting the number of words. Repeated words were scored only once and derived words were excluded [14].

In the oral diadochokinesia test, participants were asked to say the /pataka/ sequence as fast as they could. The emission was recorded and analyzed in Praat software (publicly available on web). The variable syllables/second was based on the number of syllables emitted in the first eight seconds.

### 2.3. Statistical Analysis

Data showed normal distribution (tested by Kolmogorov-Smirnov). Student's *t*-tests compared age and years of formal education of PD and control groups. Chi-square tests investigated sex distribution differences. Analyses of variance (ANOVA) were performed to compare executive function, verbal fluency, and oral diadochokinesia (considered as dependent variables) of both groups.

Pearson correlation tests examined possible correlations between executive function, verbal fluency, and oral diadochokinesia in PD group. Coefficients higher than 0.799 were considered as strong and coefficients between 0.400 and 0.799 were considered as moderate [19]. Fisher's test was used to compare correlation coefficients. In all tests, the level of significance was set at alfa < 0.05.

## 3. Results

Demographic characteristics are displayed in Table 1. The groups did not significantly differ in age, gender, years of formal education, and Mini-Mental State Examination scores (Table 1). Fifteen participants were classified as Hoehn and Yahr 2, twelve as 2.5, and thirteen as 3.

### 3.1. Trail Making Test

**Figure 1:**
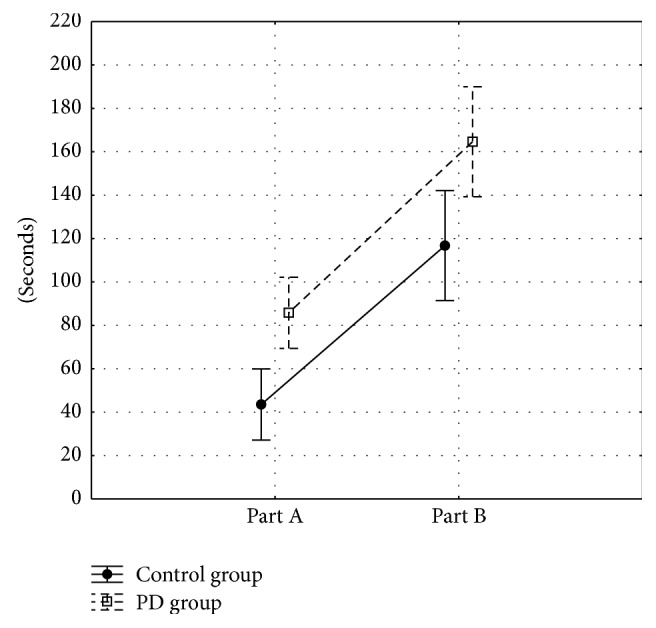
Performance on parts A and B of Trail Making Test (TMT).

People with PD needed more time to complete parts A and B of TMT than controls. ANOVA showed significant differences between groups (*F*_1,78_ = 10.55; *p* = 0.002) and between TMT parts (*F*_1,78_ = 154.02; *p* < 0.001). Part B showed longer times than part A. TMT delta did not significantly differ between the groups (*p* = 0.855). No interaction between groups and parts was observed (*F*_1,78_ = 0.20; *p* = 0.652) (Figure 1).

### 3.2. Verbal Fluency Tests

**Figure 2:**
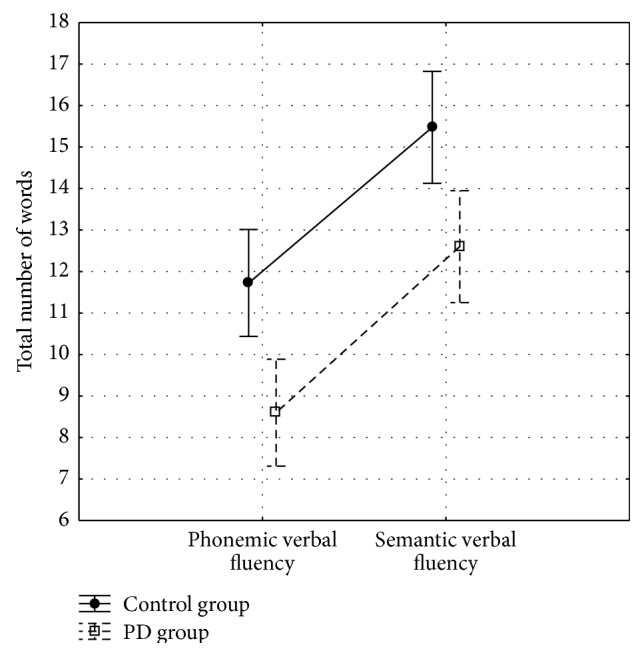
Performance on phonemic and semantic verbal fluency tests.

People with PD said fewer words in both fluency tests, compared to controls. ANOVA showed a significant difference between groups (*F*_1,78_ = 12.98; *p* < 0.001). People in PD group said fewer words in the phonemic fluency than in the semantic fluency test. ANOVA showed a significant difference between fluency tests (phonemic or semantic) (*F*_1,78_ = 81.23; *p* < 0.001). No interaction between groups and tests was observed (*F*_1,78_ = 0.84; *p* = 0.772) (Figure 2).

### 3.3. Oral Diadochokinesia Test

**Figure 3:**
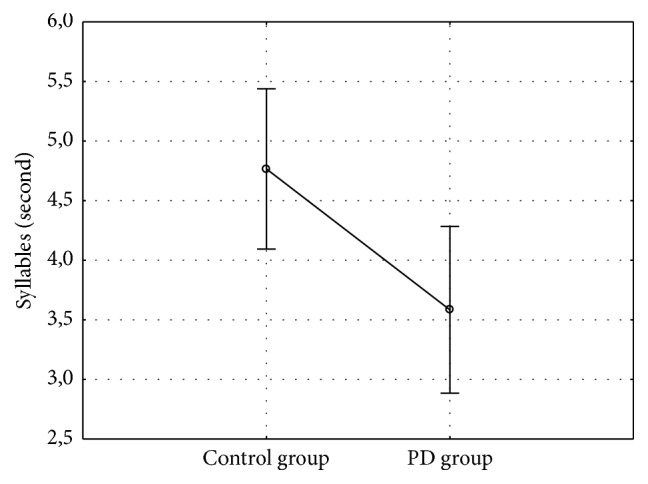
Performance on oral diadochokinesia test.

People with PD repeated the sequence /pataka/ less times than controls (*F*_1,23_ = 6.36; *p* = 0.019) in 8 seconds (Figure 3).

### 3.4. Correlation Analysis

Pearson correlation tests investigated possible relationships between the times on part A, part B, and delta of TMT, the number of words said on phonemic/semantic verbal fluency tests, and the number of syllables repetitions in oral diadochokinesia test. The correlations are displayed in Table 2. Moderate-to-strong correlations were found between TMT, phonemic verbal fluency, and oral diadochokinesia (Table 2).

The correlation coefficients were compared by Fisher's test and they are displayed in Table 3. The correlation between part B of TMT and phonemic verbal fluency was significantly stronger than the correlation between part A of TMT and phonemic verbal fluency (*p* = 0.050). The correlations between parts A and B of TMT and oral diadochokinesia were significantly stronger than the correlation between delta of TMT and oral diadochokinesia (*p* = 0.011 and *p* = 0.018, resp.) (Table 3).

## 4. Discussion

The present study compared the performance of people with PD with a control group on executive function, phonemic/semantic verbal fluency, and oral diadochokinesia tests.

People with PD showed more difficulty on TMT than controls. Both PD and control groups showed poorer performance in part B of TMT, compared to part A. Part A evaluates motor speed and coordination. Part B requires the same motor control but demands more complex cognitive skills (mental flexibility, visual scanning, and response inhibition) [10]. Part B of TMT can detect mild cognitive impairment in people with PD [20] and predict difficulties in instrumental activities of daily living [21].

In the verbal fluency test, people with PD said fewer words than controls, especially in the phonemic test. Previous studies showed that the phonemic verbal fluency was impaired in people with PD, due to the association between substantia nigra volume and phonemic verbal fluency [22]. Besides, as nouns are stored by temporal lobe neurons, the semantic verbal fluency would be more affected in people with temporal lobe lesions, for example, Alzheimer's disease [23], than in people with PD. People with PD show higher preservation of semantic content pathways and usually rely on semantic cues to facilitate lexical search [24]. In the present study, both groups showed better performance on semantic verbal fluency. Although verbal fluency can be affected in people with a low educational status or mild cognitive impairment, animals' names are one of the easiest semantic categories. People are exposed to this kind of information since childhood, which can explain our findings.

People with PD said less syllables/second than controls in oral diadochokinesia. In dual tasks with motor and cognitive demands, patients with PD have difficulty in both components (e.g., gait [20] and alternating steps [21]). This can also be observed in oral diadochokinesia. Another possible explanation would be the difficulty of patients with PD to find a speed-accuracy trade-off in repetition tasks of sequences, as in /pataka/. The speed may have been prioritized over the accuracy [25]. People with PD who are pressing for performance speed may have taken longer to resolve the interference (e.g., differentiate /pa/ versus /ta/ versus /ka/) that arises from the activation of an unintended response.

The present study investigated the relationships between executive function, verbal fluency, and oral diadochokinesia. We hypothesized that verbal fluency performance would show higher correlation with part B of TMT (which is also a cognitive-motor measure) than with part A of TMT (motor measure) and delta TMT (cognitive measure). A strong correlation between phonemic verbal fluency and part B of TMT was found. Both phonemic verbal fluency and executive function are related to caudate nucleus circuitry integrity [1, 22]. Dopamine depletion in the basal ganglia affects the main connections with the frontal lobe and compromises the activation of two major regions of projection: premotor areas (e.g., supplementary motor area, responsible for motor planning) and frontal lobe dorsal and ventral regions (involved in cognitive abilities) [1]. Semantic processing involves the activation of cortical areas (including motor areas) and depends on the engagement of the left frontal cortex [26, 27]. When the neural projections to all these cortical areas are lesioned, people with PD may show difficulties in motor planning and semantic processing.

We expected the correlation between the phonemic verbal fluency and part B of TMT to be the strongest and this hypothesis was confirmed. Fisher's test showed that the correlation between part B of TMT and phonemic verbal fluency was significantly higher than the correlations between phonemic verbal fluency and the other scores of TMT (part A and delta). The strong correlation between part B of TMT and phonemic verbal fluency suggests that dual-task performance is important in both tasks. The motor components of tracing the trail (part B of TMT) and speaking (phonemic verbal fluency) may be competing for resources that would be allocated exclusively to the cognitive components (e.g., following the sequence in part B of TMT and recalling the words in phonemic verbal fluency).

Few studies consider that, besides reflecting cognitive impairment, verbal fluency is also influenced by motor control [11, 24]. Verbal fluency demands motor coordination, speed, misspelled words inhibition, and mental flexibility for word selection. Interestingly, the semantic verbal fluency did not show the same correlation with part B of TMT. This finding agrees with Gurd (2000) [28] who showed that the motor component did not affect semantic and phonemic fluency tasks in the same way. Therefore, semantic and phonemic tasks should be combined in the evaluation of people with PD, because they show distinct levels of difficulty. PD and control groups showed better performance in the semantic fluency test. This fact can explain why semantic fluency scores did not correlate to other variables.

We hypothesized that oral diadochokinesia would show higher correlations with part A of TMT (motor measure) than with part B (cognitive-motor measure) and delta of TMT (cognitive measure). Diadochokinesia scores were strongly correlated to part A of TMT and to phonemic verbal fluency scores. This correlation was expected, since both tests measure motor speed. Part A of TMT is influenced by motor coordination and verbal fluency depends on quickly producing syllables.

The correlation between oral diadochokinesia and part B of TMT was strong, contradicting our hypothesis. The correlations between oral diadochokinesia and parts A and B of TMT were significantly higher than the correlation with TMT delta. However, correlation coefficients were not significantly different when compared to each other by Fisher's test. Therefore, oral diadochokinesia (with /pataka/ syllables) cannot be considered an exclusively motor task. Oral diadochokinesia is a cognitive-motor task. The switching syllables from /pa/ to /ta/, from /ta/ to /ka/, and from /ka/ to /pa/ involve not only motor control but also inhibition control, task switching, and sequencing, as part B of TMT. Also, in tasks demanding speed and accuracy, people with PD tend to have poor accuracy when asked to focus on speed, which can be attributed to the flaw on inhibitory control [25]. These facts also explain the moderate correlation between oral diadochokinesia and TMT delta, which is a cognitive measure.

The present study shows that the cognitive and motor interference that can be observed in complex tasks as gait and balance [29, 30] can also be observed in verbal fluency and oral diadochokinesia. Our findings amplify the knowledge of dual-task paradigm in people with PD: cognitive-motor interference also occurs in speech production (verbal fluency and oral diadochokinesia) and paper and pencil tests (TMT).

These new facts lead to reflections on how to interpret the results of verbal fluency and oral diadochokinesia. It is important to consider that cognitive and motor overload may be caused when multiple cognitive and motor components are performed simultaneously. The increase in cognitive-motor demands can impair postural stability and gait in PD [31]. Therefore, the positioning (sitting versus standing) during verbal fluency or oral diadochokinesia assessment may also influence the results, as standing requires higher motor control than sitting. If the motor aspects of cognitive-motor tasks are not fully considered, cognitive impairment can be overestimated in people with PD.

We must mention that only moderately affected participants were evaluated (Hoehn and Yahr 2-3: 27 classified as 2 and 2.5). Therefore, it is important to note that these analyses cannot be generalized to all PD severities. We used TMT as the executive function measure, but there are other tests that can be used for more detailed cognitive assessment. We used words with F and animals as phonemic and semantic verbal fluency measures, but there are other tests that can be used for more detailed verbal fluency evaluation. Many PD and control participants had low educational status (mean: 10.5 years of formal education). Education affects the performance on all tasks of the present study [32, 33]. Future studies should investigate the influence of PD severity and education on executive function, verbal fluency, and oral diadochokinesia. Although the two groups did not have significant differences in age and education, future studies should pair PD and control volunteers by age and education.

## 5. Conclusion

People with PD showed more difficulty than controls in executive function, semantic and phonemic verbal fluency, and oral diadochokinesia. Parts A and B of TMT correlated to phonemic verbal fluency and to oral diadochokinesia. This cognitive-motor interaction in verbal fluency and oral diadochokinesia must be considered not to overestimate the cognitive or motor impairments in people with PD.

## Figures and Tables

**Table 1:** Demographic data.

Groups	People with PD	Controls	*p* value
Age (years)	67.2 ± 4.3	67.0 ± 7.9	0.472
Education (years)	9.8 ± 4.9	11.2 ± 4.5	0.088
Gender (F/M)	24/16	27/13	0.485
Mini-Mental State Examination score	27.7 ± 2.1	27.8 ± 1.3	0.290
Disease duration (years)	9.3 ± 6.1	—	—
UPDRS III	27.9 ± 12.0	—	—

PD: Parkinson's disease; UPDRS III: Unified Parkinson's Disease Rating Scale-motor section [17].

**Table 2:** Correlations between executive function (parts A and B and delta of Trail Making Test), phonemic/semantic verbal fluency, and oral diadochokinesia scores in people with Parkinson's disease (Pearson correlation coefficients).

	Part A (TMT)	Part B (TMT)	Delta (TMT)	Oral diadochokinesia
Phonemic verbal fluency test	*r* = − 0.712 *p* = 0.009^*∗*^	*r* = − 0.874 *p* = 0.001^*∗*^	*r* = − 0.740 *p* = 0.006^*∗*^	*r* = 0.684 *p* = 0.014^*∗*^

Semantic verbal fluency test	*r* = − 0.311 *p* = 0.325	*r* = − 0.468 *p* = 0.125	*r* = − 0.339 *p* = 0.281	*r* = 0.325 *p* = 0.303

Oral diadochokinesia	*r* = − 0.838 *p* = 0.001^*∗*^	*r* = − 0.824 *p* = 0.001^*∗*^	*r* = − 0.689 *p* = 0.013^*∗*^	—

^*∗*^ *p* < 0.05; TMT: Trail Making Test.

**Table 3:** Comparison between correlation coefficients (Fisher's tests).

	Part B X phonemic verbal fluency (*r* = −0.874)	TMT delta X phonemic verbal fluency (*r* = −0.740)
Part A X phonemic verbal fluency (*r* = −0.712)	0.050^*∗*^	0.780
TMT delta X phonemic verbal fluency (*r* = −0.740)	0.100	—

	Part B X diadochokinesia test (*r* = −0.824)	TMT delta X diadochokinesia test (*r* = −0.689)

Part A X diadochokinesia test (*r* = −0.838)	0.850	0.011^*∗*^
Part B X diadochokinesia test (*r* = −0.824)	—	0.018^*∗*^

^*∗*^ *p* < 0.05.
